# Microwave-Assisted Fabrication of High Energy Density Binary Metal Sulfides for Enhanced Performance in Battery Applications

**DOI:** 10.3390/nano13101599

**Published:** 2023-05-10

**Authors:** Kenna L. Salvatore, Justin Fang, Christopher R. Tang, Esther S. Takeuchi, Amy C. Marschilok, Kenneth J. Takeuchi, Stanislaus S. Wong

**Affiliations:** 1 Department of Chemistry, State University of New York at Stony Brook, Stony Brook, NY 11794-3400, USA; 2 Department of Materials Science and Chemical Engineering, State University of New York at Stony Brook, Stony Brook, NY 11794-3400, USA; 3 Institute for Energy Sustainability and Equity, State University of New York at Stony Brook, Stony Brook, NY 11794-3400, USA; 4 Interdisciplinary Science Department, Brookhaven National Laboratory, Upton, NY 11973-5000, USA

**Keywords:** binary metal sulfides, microwave chemistry, synthesis, battery applications

## Abstract

Nanomaterials have found use in a number of relevant energy applications. In particular, nanoscale motifs of binary metal sulfides can function as conversion materials, similar to that of analogous metal oxides, nitrides, or phosphides, and are characterized by their high theoretical capacity and correspondingly low cost. This review focuses on structure–composition–property relationships of specific relevance to battery applications, emanating from systematic attempts to either (1) vary and alter the dimension of nanoscale architectures or (2) introduce conductive carbon-based entities, such as carbon nanotubes and graphene-derived species. In this study, we will primarily concern ourselves with probing metal sulfide nanostructures generated by a microwave-mediated synthetic approach, which we have explored extensively in recent years. This particular fabrication protocol represents a relatively facile, flexible, and effective means with which to simultaneously control both chemical composition and physical morphology within these systems to tailor them for energy storage applications.

## 1. Introduction

Binary semiconducting metal sulfides (M_x_S_y_) possess novel optical, electrical, and chemical properties, and have been considered for a multitude of different applications. Specifically, they have been developed as building blocks for photovoltaic devices, including dye-sensitized cells, all-inorganic nanoparticle solar cells, and hybrid nanocrystal-polymer composite solar cells, in addition to lasers, waveguides, and other optoelectronic devices [1,2,3,4]. Moreover, they have found usage as either supercapacitors or catalysts for the hydrogen and oxygen evolution reactions [5,6,7]. In addition, they have been investigated for biomedical applications [8,9,10], including but not limited to biosensors and photothermal therapy.

In the context of this Review, metal sulfides frequently have been assessed for their viability as components of batteries. Specifically, they can function as conversion materials, similar to that of analogous metal oxides, nitrides, or phosphides, and are characterized by their high theoretical capacity and correspondingly low cost [11,12]. In particular, binary metal sulfides are attractive candidates as electrodes for battery applications, because of their advantageous attributes for increasing latent charge and ion mobility through (i) the exposure of reactive surface areas, (ii) a favorable reduction in ion diffusion distances, and (iii) an enhancement in cyclability. Furthermore, the utilization of metal sulfides as battery-active materials is particularly tantalizing not only due to the possibility of both anionic and cationic redox activity, where the transition metal cation can be reduced, but also because of the inclusion and functionality associated with the sulfur moiety [13,14]. Accessing the electrochemical reactivity of both species necessitates employing a broad voltage range; however, the benefit of this is the possibility of very high capacities. The use of a wide voltage window renders the selection of the current collector critical. For lithium batteries, low voltages can only be accessed using a copper current collector to avoid the formation of lithium-aluminum alloys with an aluminum foil current collector [15]. However, to access high voltages, the use of copper is not possible, due to the possibility of copper oxidation. A solution that has been employed in the past has been to use a carbon matrix as the current collector in order to avoid the reactivity that is inherent to the metal foil current collectors [16]. By comparison with more commonly used oxides, metal sulfides benefit from (a) a wider palette of possible achievable redox chemistries, (b) superior conductivities, and (c) improved reversibilities [17]. Conversely, sulfides are hindered from realizing their full theoretical potential for battery applications by limitations such as poor cycling retention due to issues ascribable in part to volume expansion and low conductivity.

One promising approach towards mitigating these deficiencies is to produce these materials as their nanoscale analogs [18]. As is well known, nanomaterials can possess properties which differ significantly from and can be better than those of the corresponding bulk due to the vast increase in surface area-to-volume ratios and associated surface-induced effects at this scale. Specifically, the advantages of creating nanoscale motifs include but are not limited to (a) an increase in the number of active sites, (b) shorter distances associated with a more rapid and effective electron and ion transport, (c) the potential for spatial confinement and control of electron and ion movement in either one or more dimensions, and (d) a greater resistance to volume change (and hence, an improved cycle stability).

Indeed, the community has found that, while shrinking down metal sulfides to nanoscale formulations is a helpful generalizable strategy, the approach is complicated by factors such as (1) dimensionality and (2) the presence of conductive additives, which also play key roles in terms of impacting the inherent electrochemical performance of these materials. We define the nature of dimensionality in terms of the conventionally accepted sense of enabling spatial confinement along orthogonal planes of as-prepared structures to the nanoscale regime. Hence, representative examples of zero-dimensional (0D), one-dimensional (1D), two-dimensional (2D), and three-dimensional (3D) nanomaterials include quantum dots, nanowires, nanosheets, and sea-urchin assemblies, respectively.

From the literature, it is known that 0D materials tend to maintain not only a greater structural and cycling stability but also a reduced volume expansion compared to bulk. By comparison, 1D structures exhibit not only an increased surface area but also shortened distances for both electron and Li-ion transport. In addition, 2D materials are characterized by large surface areas, thereby resulting in an increased number of exposed active sites, shortened Li-ion diffusion distances, and superior structural stability [11]. However, despite the clear benefits associated with 0D, 1D, and 2D motifs, the performance of these materials can often be hindered by aggregation effects, which manifest themselves in observables such as a decrease in cycling [19]. Hence, 3D architectures, which ironically often consist of agglomerations of individual 0D, 1D, and/or 2D components [11], have been found in some cases to combine and incorporate the best attributes of all of these constituent units. Specifically, 3D motifs often display a high overall surface area for reactivity, especially when compared with either 0D or 1D analogs, with implications for (a) the presence of a plethora of active surface sites; (b) diminished Li diffusion distances; (c) an enhanced structural stability; and (d) a favorable reduction in not only overall aggregation of the material but also undesirable re-stacking of lower-dimensional building blocks, such as nanosheets [11]. As such, 3D nanoscale structures frequently demonstrate better structural stability, specific capacity, and rate performance metrics as compared with 0D, 1D, and 2D analogs, respectively, due not only to their increased reactive surface areas but also to their ability to moderate volume expansion effects during relevant redox reactions [20,21,22,23].

With respect to the addition of conductive additives, we note that although a number of other equally valid and significant approaches (including but not limited to either the insertion of molecular and elemental dopants or the addition of self-healing polymers [24,25]) also exist, herein we focus our discussion on the introduction of discrete carbon-based materials, such as 1D carbon nanotubes (CNTs), 2D graphene, graphene oxide (GO), reduced graphene oxide (rGO), and liquid-exfoliated graphene (LEGr). It has been noted that these materials, in combination with either metal sulfides or oxides, lead to unique hierarchical assemblies, characterized by a greater stability, a notably better conductivity, and the capability for accommodating volume changes during cycling [26]. As an example, analysis of a mixture of multi-walled carbon nanotubes (MWNTs) coupled with metal oxides revealed notable improvements in electrochemical activity for battery applications as compared with pristine and unfunctionalized metal oxides alone [27]. Furthermore, other reports have emphasized the benefits of introducing carbon additives, such as rGO [28,29] and graphene [30,31], for appreciably boosting cycling stability and capacity.

With the ultimate goal of enhancing conductivity and reducing the extent of volume change due to electrochemical reactions within binary metal sulfides, this review therefore centers on structure–composition–property relationships, emanating from deliberative attempts to either (1) vary and alter the dimension of nanoscale architectures or (2) introduce conductive carbon-based entities. It should be noted that many distinctive synthesis techniques [32,33,34], such as hydrothermal, solid-state, and solvothermal-inspired reactions, have been previously reported for these nanoscale metal sulfide motifs. Nevertheless, as compared with these other methods, rapid and uniform heating (made possible using microwave irradiation in particular) can yield advantages, such as a short reaction time, a facility of synthesis, and a consistency of the resulting product in terms of size, shape, and composition.

Therefore, in this Review, we will primarily concern ourselves with metal sulfide nanostructures generated by a microwave-mediated synthetic approach [35,36], which we have explored extensively in recent years. To highlight the significance of this body of work, in Table 1, we summarize data from the literature pertaining to specific capacities measured over multiple cycles of diverse metal sulfide electrode nanomaterials, synthesized using microwave-assisted processes. The metal sulfide nanoparticles we have considered in this Review, namely copper sulfide, iron sulfide, molybdenum sulfide, and vanadium sulfide, can be broadly divided into two general categories, based on their crystal structure, i.e., layered versus non-layered. In this context, we consider and correlate some of the more important defining parameters of these systems, such as theoretical capacity, cost, toxicity, and morphology, as well as the facility of their synthesis.

## 2. Advantages and Disadvantages of Microwave-Assisted Methods

The obvious question is: why microwave-based chemistry [55]? Microwave-assisted methods to produce metal sulfides represent the use of a potentially environmentally sustainable means with which to fabricate nanomaterials using shortened reaction times with uniform heating across the reaction medium, while simultaneously maintaining control over a large number of reaction parameters in order to enable the facile production of respectable quantities of pure and homogeneous products with high yields. Many wet chemical processes are unable to readily satisfy all of these criteria at once, associated with ensuring sample purity. Moreover, whereas reaction variables, such as time, temperature, and precursors, can be carefully tuned and tailored, as with most other synthesis protocols including but not limited to hydrothermal and solvothermal techniques, microwave-derived procedures [35,36] offer a much broader parameter space with which to tweak and optimize product formation with comparative ease, including but not limited to power, pressure, and solvent selection.

In particular, solvent identity is a crucial choice, because the solvent must not only be miscible with the precursor solute molecules but also be capable of absorbing microwave energy, as measured by its tangent factor (δ) [56]. As an example, in a previous paper by this group, we found that VS_4_ nanoflowers could be reliably generated over a range of sizes in high quantities and yields simply by changing the polarity of the solvent, which in turn influenced the resulting reaction kinetics and thereby impacted the dimension and morphology of the final product [18].

Prior reports have found that the choice of the synthesis method used to create testable samples (such as metal sulfides in particular) has a significant impact, with clear implications for the resulting electrochemical performance. As an illustrative example comparing the relative benefits of using materials derived from microwave-assisted versus hydrothermal methods, several studies have analyzed this issue in the context of optoelectronic applications [36], supercapacitors [57], electrocatalytic reactions such as the hydrogen evolution reaction (HER) [58], and lithium-ion batteries (LIBs) [59,60,61]. In particular, with respect to the performance of ZnCo_2_O_4_ flower-like materials [8] generated using both synthesis techniques, the sample fabricated via microwave irradiation exhibited an enhanced cycling performance after 45 cycles of 1411 mA h g^−1^ as compared with the much lower value of 217 mA h g^−1^ for its hydrothermally produced analogue. One plausible explanation for the observed difference is that the microwave-initiated material in question was characterized by smaller crystallite sizes. As we will subsequently explore, similar types of behavior have been observed with respect to the use of distinctive classes of metal sulfides incorporated within lithium-ion, sodium-ion, magnesium-ion, and lithium-sulfur batteries as either the anode or the cathode.

For the sake of completeness, we should note that one possible disadvantage of microwave-assisted methods based on anecdotal evidence in our lab is that, depending on the targeted system, sample size, shape, and crystallinity are not necessarily homogeneous and uniform within isolated samples produced by this technique, and as such, are more difficult to simultaneously control. Moreover, depending on the configuration of the microwave reactor, the maximum volume of a reaction vessel may be limited, thereby potentially hindering scale-up of the method. As a corollary issue [62], the fact that microwave systems cannot easily be incorporated into existing engineering scaffolds for the fabrication of large-scale materials needed for battery production is a significant disadvantage, since the replacement of current similar conventional systems would require a good deal of investment. Additionally, in many cases involving the use of microwave irradiation in facilitating chemical transformations, the true origins of the overall microwave enhancement and heating effect are either uncertain or poorly understood, which does not help in enabling the widespread implementation and acceptance of this technology.

## 3. Non-Layered Binary Sulfides (Copper, Zinc, Cobalt, Indium, and Iron)

We will first consider binary metal sulfides characterized by non-layered crystal structures, i.e., typically sulfides incorporating late-transition and post-transition metals. Many types of these metal sulfides generated via microwave-assisted synthesis have been utilized for battery applications. One common example is green covellite copper sulfide (CuS), which possesses an elevated electronic conductivity (10^−3^ S cm^−1^), a favorable theoretical capacity (560 mA h g^–1^), and a correspondingly large voltage hysteresis profile—of relevance for their use as LIB anodes [63,64]. Not surprisingly, due to its comparatively low cost and high abundance, CuS has been tested in magnesium-ion [37,65,66], lithium-ion [26,39,40,67,68], and sodium-ion [38,41] battery applications. In terms of dimensionality, CuS spheres [39], nanotubes [66], nanosheets [37,38,65,68], nanodiscs [69], and nanoflowers [40] have been generated using microwave-mediated processes.

**Figure 1:**
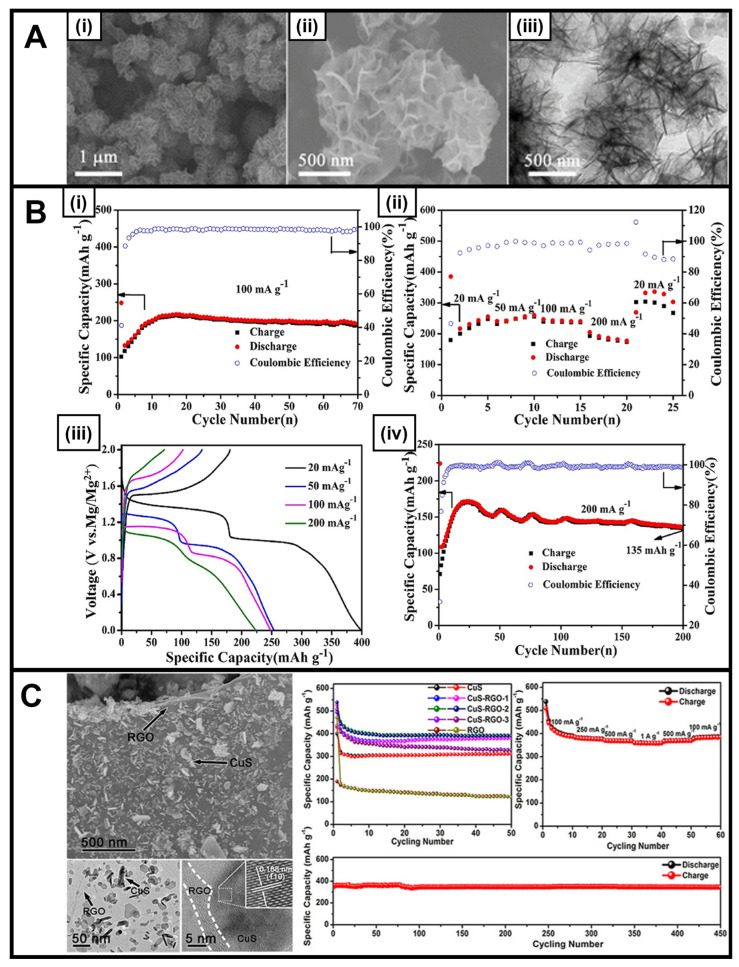
(**A**) (**i**,**ii**) SEM images and (**iii**) TEM image of the hierarchical CuS nanosheets, produced via a ‘microwave heating’ method [37]. (**B**) (**i**) Capacity and CE of the CuS electrode at 100 mA g^−1^, (**ii**) rate capabilities of the CuS electrode with various current densities from 20 to 200 mA g^−1^, (**iii**) discharge and charge profiles at different current densities, and (**iv**) long-term cycling of the CuS electrode at 200 mA g^−1^ [37]. (**C**) FESEM and TEM images of CuS-rGO-2 at low and high magnifications and cycling performance of CuS, CuS-rGO-1, CuS-rGO-2, CuS-rGO-3, and rGO at a current density of 100 mA g^−1^; rate performance of CuS-rGO-2; and long cycling performance of CuS-RGO-2 at 1 A g^−1^ [41]. Panels (**A**,**B**) have been adapted with permission from ref. [37]. Copyright 2019 American Chemical Society. Panel (**C**) has been adapted with permission from ref. [41]. Copyright 2017 American Chemical Society.

In a typical reaction, copper chloride (copper source) and thioacetamide (sulfur source) were added to a mixed solvent containing water and ethanol and irradiated at 300 W for 30 min to yield nanosheets measuring roughly 200 nm in diameter (Figure 1A) [37]. These materials exhibited promise as Mg-ion cathodes, demonstrating not only an enhanced reversible discharge capacity of 300 mA h g^–1^ at 20 mA g^–1^ but also an exceptional cycling stability of 135 mA h g^–1^ at 200 mA g^–1^ over 200 cycles (Figure 1B). The performance of these motifs can be attributed to their 3D hierarchical structure, in which the constituent component nanosheets maintain a large surface area that is exposed for interaction with the electrolyte while also providing sufficient space for expansion and contraction, which is associated with ion intercalation and removal. Furthermore, as another example, camellia-like nanosheet superstructures of CuS have been produced via the combination of PVP, copper chloride, and sodium thiosulfate through a microwave-assisted reaction [38]. The resulting catalyst served as an excellent anode material in sodium-ion batteries, exhibiting a stable capacity of 347.1 mA h g^−1^, even after 1000 cycles at 5.0 A g^−1^ [38].

Reflecting an alternate approach, CuS materials have also been combined together with conductive carbon additives, such as CNTs [39,67], reduced graphene oxide (rGO) [40,41], and graphene [26], in order to boost both cycling stability and overall conductivity versus control samples consisting of CuS alone; the incorporation of reduced graphene oxide in particular led to noticeable benefits. We elaborate on these as-produced structures as follows. A CuS/CNT composite was generated via an in situ microwave-assisted reaction, in which CuS spheres were grown onto the underlying CNT backbone; indeed, the nucleation of CuS on the CNTs was enabled by the fact that as a material, the CNTs preferentially heated faster than the solvent itself under microwave irradiation conditions [39].

Moreover, at different levels of CNT incorporation, the microwave-generated CuS/CNT composite performed noticeably better as an anode in a Li-ion battery than identical analogs derived from ball milling processing, an observation which can be ascribed to an improved integration between CuS and CNTs within microwave-generated samples. The electrochemical testing was conducted using an electrode prepared on a Cu foil positioned within a coin cell versus a lithium counter electrode using 1 M lithium bis(trifluoromethanesulfonyl)imide within a 1,3-dioxolane and dimethyl ether electrolyte. As such, the cycling tests were conducted in lithium metal half-cells. Specifically, in these microwave-derived composites, excellent stability was recorded after 450 cycles at 400 mA h g^−1^, with measured capacities ranging from 437 to 569 mA h g^−1^ as a function of increasing CNT content.

A complementary CuS-rGO composite consisting of CuS nanosheets and rGO was tested as an anode replacement for Na-ion storage. It yielded a measured specific capacity of 392.9 mA h g^–1^ after 50 cycles at a current density of 100 mA h g^–1^ coupled with a high initial Coulombic efficiency of 94% (Figure 1C), in addition to a sustained specific capacity of 345 mA h g^–1^ noted after 450 cycles at a current density of 1 A g^–1^ [41]. The effects of the electrolyte were also tested within this system, with demonstrably improved performance measured in an ether-based solvent due to its ability to suppress polysulfide intermediates. A final example of a CuS/graphene composite was created under in situ reaction conditions involving the use of sodium thiosulfate as the sulfur precursor and copper chloride as the copper precursor in a mixture, which was subjected to a power cycling algorithm; the as-produced CuS nanoparticles were deposited onto underlying graphene sheets. The resulting composite gave rise to an improved reversible capacity of 348 mA h g^−1^, which was maintained after 1000 cycles at a current density of 2.0 A g^−1^. It was determined that the CuS component had benefited from enhanced Li-ion transfer and reduced contact resistance, as a result of its attachment to adjoining graphene sheets [26].

Other studies have explored additional, analogous materials, such as zinc sulfide (ZnS) [42], indium sulfide (In_2_S_3_) [44], nickel sulfide [45], and cobalt sulfide (Co_9_S_8_) [43], as well as iron sulfide (FeS_2_ and Fe_3_S_4_) for sodium-ion [42,43,45] and lithium-ion battery [44,70] applications. It is worth noting that ZnS is known for a favorable combination of non-toxicity, comparatively low cost, and relatively high theoretical capacity in LIB (962.3 mA h g^−1^) [71]. As an example, ZnS nanoparticles were made via a microwave-assisted method in less than 15 min in the presence of varying amounts of GO [42]. By optimizing the amount of GO within the sample, the ensuing ZnS-rGO electrode delivered a high specific capacity of 481 mA h g^−1^, measured at 100 mA g^−1^ after 50 cycles for sodium-ion battery anodes, denoting observations ascribable in part to the greater surface area and better electronic conductivity associated with the addition of rGO [42].

Co_9_S_8_ materials possess an intrinsically high capability, when used as anode materials. To reinforce the significance and advantages of graphene addition, composites incorporating quasi-spherical motifs of microwave-derived Co_9_S_8_ and rGO delivered improved activity within SIB anodes. Specifically, a high reversible capacity of 426.2 mA h g^−1^ at a current density of 100 mA g^−1^ was measured and remained at a relatively high value of 346.3 mA h g^−1^ even after 30 cycles, a finding which was attributable in part to the presence of the rGO additive. The presence of this additive reduces the Co_9_S_8_ particle size from 200–400 nm down to ~20 nm, buffers volume changes associated with charging and discharging, and increases conductivity [43].

**Figure 2:**
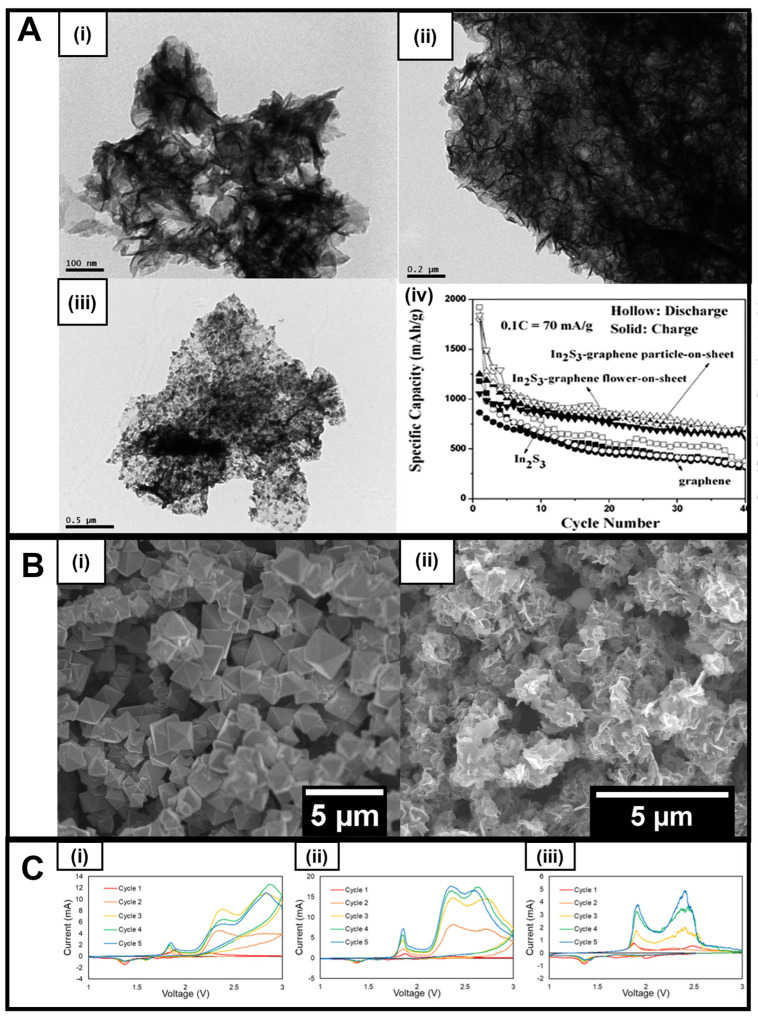
(**A**) (**i**) TEM image of In_2_S_3_ products prepared at 160 °C after 20 min microwave irradiation; (**ii**) TEM image of In_2_S_3_–graphene composite (flower-on-sheet) [44]; (**iii**) TEM image of In_2_S_3_–graphene composite (particle-on-sheet); (**iv**) Cycling performance of In_2_S_3_–graphene composites at 0.1C (70 mA g^−1^) (hollow: discharge, solid: charge) [44]. (**B**) (**i**) SEM image of FeS_2_ octahedra and (**ii**) SEM image of Fe_3_S_4_ nanosheets. (**C**) Cyclic voltammetry (CV) results of (**i**) as-prepared Fe_3_S_4_, along with heterostructures created by incorporation with either (**ii**) MWNT or (**iii**) graphene. The CV data, show that the introduction of MWNTs increases measured current values as compared with either Fe_3_S_4_ alone or with the analogous graphene-containing heterostructure. Data were collected at a 0.1 mV/s scan rate with voltage limits of 1.0 to 3.0 V. Panel (**A**) has been reproduced from ref. [44] with permission from the Royal Society of Chemistry.

Additionally, while mainly utilized in the context of photocatalysts and solar cells, In_2_S_3_ is attractive for battery applications, because it possesses not only a reasonably sizeable theoretical capacity of 713 mA h g^−1^ but also a desirable spinel crystal structure similar to that of high-performing materials such as magnetite and lithium titanate [72]. Not surprisingly, In_2_S_3_ nanoflowers and nanoparticles have been dispersed onto graphene nanosheets to form a sandwich-like hierarchical structure for LIB anodes [44]. As a starting point, In_2_S_3_ nanoflowers were first optimized by controlling parameters, such as temperature and time to construct structures measuring several hundred nanometers in diameter at 140 °C for 20 min upon reaction of indium chloride and thioacetamide precursor reagents with CTAB as the surfactant in water (Figure 2A(i)).

To create the corresponding In_2_S_3_-graphene composites, an in situ reaction was utilized, in which graphene was introduced into the above mixture prior to microwave irradiation. The as-formed In_2_S_3_-graphene composites exhibited two unique morphologies, denoted as “In_2_S_3_–graphene nanoparticle-on-sheet” (Figure 2A(iv)) and “In_2_S_3_–graphene flower-on-sheet” (Figure 2A(ii)) motifs. Whereas both composites revealed more than 1.5 times the reversible capacities of the pristine control material, the “In_2_S_3_-graphene nanoparticle-on-sheet” structure, in particular, gave rise to a greater cycle stability at larger currents, an observation most likely ascribed to the higher relative quantities of graphene (whose presence can not only reduce volume change but also improve mechanical stability and conductivity) versus those of the nanoflowers present within the sample (Figure 2A(iii)).

In another example, our group has recently worked on iron sulfides (FeS_2_ and Fe_3_S_4_) that can be produced using a microwave-based technique. FeS_2_ typically occurs in two forms, namely pyrite (cubic) and marcasite (orthorhombic). However, few reports have demonstrated the ability to isolate either of these crystallographic phases in large quantities at the nanoscale [73]. Nevertheless, phase control and facet formation are important parameters to consider. For example, based on previous studies, the (111) facet (compared with others) of FeS_2_ was particularly considered to be an attractive and promising candidate for LIB anode applications; however, it is difficult to synthesize, due to its comparatively high-energy formation. Because octahedra signify one of the few shapes in which this facet is predominantly featured and exposed, we recently focused on generating pure pyrite FeS_2_ octahedra using microwave-based chemistry in the absence of either surfactants or corrosive solvent conditions so as to decrease the level of impurities and defects that might have interfered with subsequent electrochemical analyses. As such, we employed a solvent mixture of water and ethylene glycol in the presence of thioacetamide and iron chloride as the sulfur and iron precursor, respectively, to fabricate high-quality octahedra (Figure 2B(i)) using a simpler, much faster, and more efficacious protocol than previous approaches [74].

Modifications to this underlying procedure enabled us to synthesize nanosheets of greigite or Fe_3_S_4_, another iron sulfide which has had limited testing for battery applications despite possessing a relatively high theoretical capacity of 725 mA h g^–1^, arising in part from the consequences of an 8e^−^ reversible conversion reaction. Greigite is unusual in that, as a sulfide-based spinel, it retains a similar structure to magnetite. To achieve our synthetic objective, we substituted L-cysteine for the sulfur precursor and optimized the metal-to-sulfur ratio in order to control not only the chemical composition but also the resulting morphology. The driving force for a successful reaction appeared to be the choice of the sulfur precursor, as it could dictate the amount of sulfur-containing species present at any given moment within the reaction medium.

Furthermore, the as-synthesized Fe_3_S_4_ nanosheets were also combined with conductive carbon additives, specifically with MWNTs and graphene, albeit by using an ex situ method. (Figure 2B(ii)). The production of these carbon-based composites fortunately did not introduce either apparent impurities or perceptible changes in the isolated morphology. Cyclic voltammetry of the as-generated samples (Figure 2C) shows that the incorporation of carbon is beneficial to the current produced, with the MWNT-containing material evincing greater current values (Figure 2C(ii)) than either the as-synthesized Fe_3_S_4_ alone (Figure 2C(i)) or the graphene-containing heterostructure (Figure 2C(iii)). This observation thereby confirms the clear advantages of introducing conductive carbon additives in order to create high-performing iron sulfide-based composite motifs.

## 4. Layered Transition Metal Dichalcogenides (MoS_2_, WS_2_, SnS_2_)

An important subset of metal sulfides encompasses the transition metal dichalcogenides (TMDC) due to their characteristic layered structure, in which transition metal atoms are sandwiched between chalcogenides in such a way to form 2D layers that are connected to other adjacent layers by weak van der Waals forces. These layers can be used to boost electrochemical performance by taking advantage of the superior ion intercalation potential both within and between the individual, discrete layers [29]. Typical TMDC materials include but are not limited to molybdenum disulfide (MoS_2_), tungsten disulfide (WS_2_), and tin disulfide (SnS_2_).

**Figure 3:**
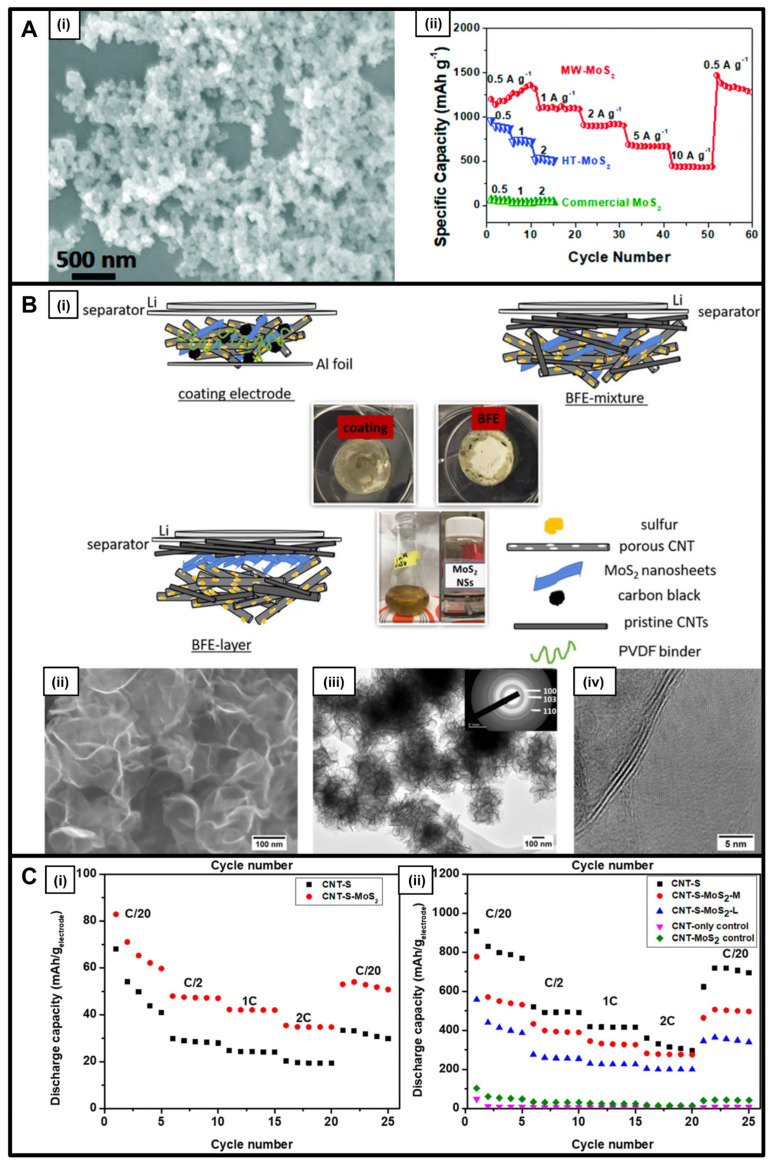
(**A**) (**i**) SEM image of MW-MoS_2_; (**ii**) Rate capabilities of MW-MoS_2_, HT-MoS_2_ and the commercial MoS_2_ at different current densities from 0.5 to 10 A g^−1^ [46]. (**B**) (**i**) Schematic illustration of coating, BFE-mixture, and BFE-layer cell configurations; (**ii**) SEM, (**iii**) TEM, and SAED pattern (inset), (**iv**) HRTEM of the as-prepared MoS_2_ nanosheets [47]. (**C**) Specific capacity versus cycle number, calculated based on sulfur weight and the total cathode weight for the coating cells (**i**) and the BFEs (**ii**) measured at 200, 400, 800, 1600, and 200 mA/g_sulfur_ discharge/charge current density [47]. Panel (**A**) has been reproduced from ref. [46] with permission from the Royal Society of Chemistry. Panels (**B**,**C**) have been adapted with permission from ref. [47]. Copyright 2019 American Chemical Society.

Molybdenum sulfide is known for its applicability as an anode replacement in lithium-ion [29,31,46], sodium-ion [28,30,31], and lithium-sulfur [47] batteries, evincing both a unique layered structure and favorable electronic properties [28]. Published reports cite the possibility of synthesizing a range of diverse morphologies, such as particles [28,46], nanosheets [30,31,47], and nanocrystals [29]. As previously implied, the choice of the synthesis protocol has an inordinate impact upon the dimensionality and architecture of the resulting product. In this vein, microwave-based methods yielded small 0D nanoparticles (‘MW-MoS_2_’) measuring 20 to 30 nm in diameter, whereas analogous hydrothermal techniques produced larger 3D flower-like motifs (HT-MoS_2_) (Figure 3A) [46]. Upon incorporation into LIB anodes, compared with these flower-like analogs, the smaller MW-MoS_2_ nanoparticles demonstrated not only an enhanced initial capacity of 1199 mA h g^−1^ but also a high stability during the cycling process, characterized by a Coulombic efficiency of 75% and an exceptional rate capability (Figure 3A) [46]. This unexpected finding was ascribed to the comparatively larger exposed surface area coupled with the substantial pore volume of samples generated during the microwave treatment versus those that had been synthesized hydrothermally.

Separately, 2D MoS_2_ nanosheets produced via a microwave-assisted method in the presence of an ether solvent were probed as cathodes within a binder-free electrode configuration (BFE) for lithium-sulfur battery applications (Figure 3B) [47]. Specifically, these MoS_2_ nanosheets exhibited an excellent capacity of 694 mA h/g_sulfur_ and a specific energy of 1435 Wh/kg_sulfur_ (600 Wh/kg_electrode_)—measured at a C/2 rate of 835 mA g^–1^ after 200 cycles within a di(propylene glycol) dimethyl ether (DPGDME) electrolyte (Figure 3C) [47]. It should be noted that the use of the BFE configuration gave rise to a significant cycling stability compared with that measured for coated electrodes. Moreover, the addition of a CNT-based network to these MoS_2_ nanosheets led to increased polysulfide trapping, which consequentially improved ion transfer and diffusion.

**Figure 4:**
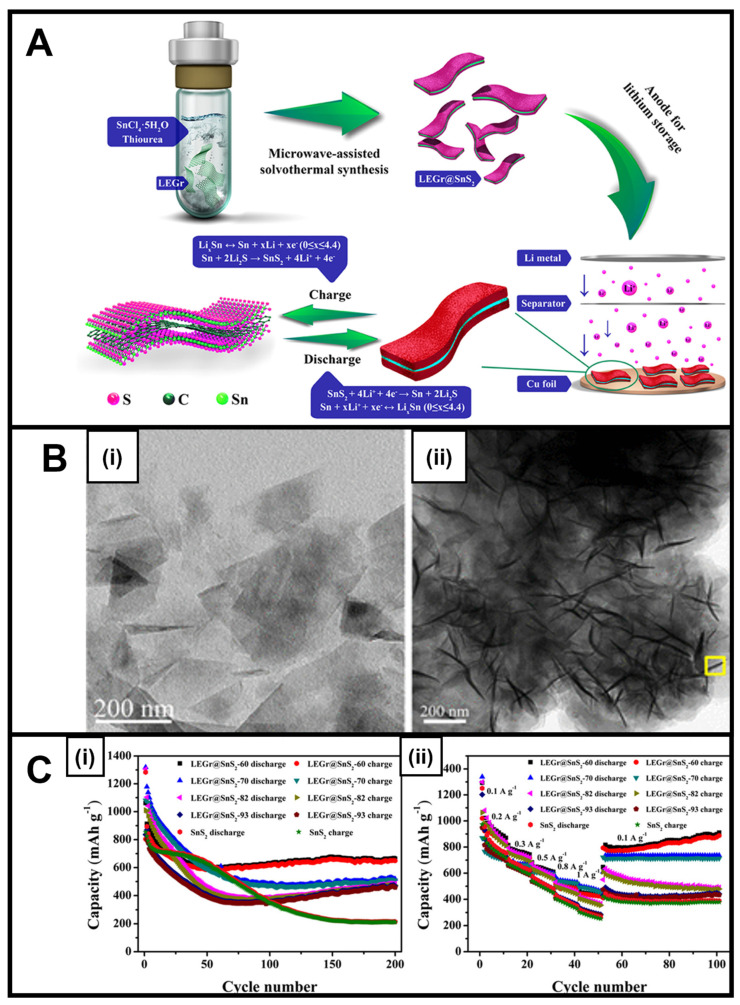
(**A**) Schematic of the Preparation Process and Applications of LEGr@SnS_2_ Heterojunctions. (**B**) (**i**) TEM image of the LEGr sheets and (**ii**) analogous TEM image of LEGr@SnS_2_-60. (**C**) (**i**) Cycling performance of SnS_2_ and LEGr@SnS_2_-X anodes at 300 mA g^−1^ current density and (**ii**) rate performance of SnS_2_ and LEGr@SnS_2_-*X* anodes measured at different current densities [48]. Panels (**A**–**C**) have been adapted with permission from ref. [48]. Copyright 2019 American Chemical Society.

Microwave-generated tin sulfide (SnS_2_), which is known for its inherently high electronic conductivity, has been tested for its applicability in lithium-ion [31,48,75,76,77,78] and sodium-ion batteries [31] in the guise of nanoflakes [31,75], intercalated sheets [76], and microflowers [77]. As a layered 2D motif, SnS_2_ gave rise to a relatively high theoretical capacity of 645 mA h g^–1^ for LIB applications. The corresponding SnS_2_-based composites, produced using a microwave-derived technique with tin and sulfur precursors in the presence of liquid exfoliated graphene (LEGr), led to an unusual architecture created by SnS_2_ nanosheets nucleating onto LEGr nanosheets (Figure 4A,B) [48]. This LEGr-based SnS_2_ composite was found to retain a high storage capacity coupled with an enhanced cycling stability of 664 mA h g^–1^ after 200 cycles at a 300 mA h g^–1^ current density (Figure 4C), with the measured performance attributed to the high conductivity and mechanical stabilization against the volume change provided by LEGr [48].

WS_2_ represents another TMDC material, that can form as favorable layered 2D sheets and enable not only superior ion intercalation but also enhanced measured reversibility [29,49]. As such, analogously synthesized LEGr@WS_2_ composites prepared using a microwave-assisted solvothermal method that suppresses the formation of WO_3_ impurities were characterized by a 2D hierarchical structure possessing excellent interfacial contact between the WS_2_ and the LEGr surfaces, denoting a desirable interface for promoting excellent electrochemical performance and cycling stability for LIB anode applications. Moreover, by varying the relative quantities of W and S within these composites, these LEGr@WS_2_ materials displayed not only a reversible capacity of 714 mA h g^–1^ after 100 cycles at a current density of 300 mA g^–1^ but also a stable capacity of 534 mA h g^–1^ after 450 cycles at a high current of 1 A g^–1^ [49].

## 5. Vanadium Sulfide

The vanadium sulfide system has historically been less well-studied and is somewhat more complex to control. Nevertheless, it can exist as either a layered or a non-layered form, depending on stoichiometric considerations. In particular, vanadium disulfide or VS_2_ possesses the typical TMDC layered structure, in which V is sandwiched between two S layers, and it has recently been attracting significant interest as an electrode material for energy storage applications [79]. In the process of attempting to create this material, our group was able to develop and optimize a reliable microwave-derived synthesis of a related vanadium sulfide, namely VS_4_, which possesses a structural similarity to both pyrite (FeS_2_) and vaesite (NiS_2_) [80].

Interestingly, VS_4_ itself is highly desirable for its high theoretical capacity for both SIBs and LIBs (1196 mA h g^–1^) [81]. Experimentally, it was found that patronite (VS_4_) arises in the presence of a carbon substrate due in part to the potential for favorable electron transfer, whereas in its absence, VS_2_ tends to form [82]. From a synthetic perspective, VS_4_ has been manufactured via microwave-mediated methods as 1D nanorods [18], 2D nanosheets [50,51], and 3D nanoflowers [18,51] to probe their efficacy as anodes for battery applications.

**Figure 5:**
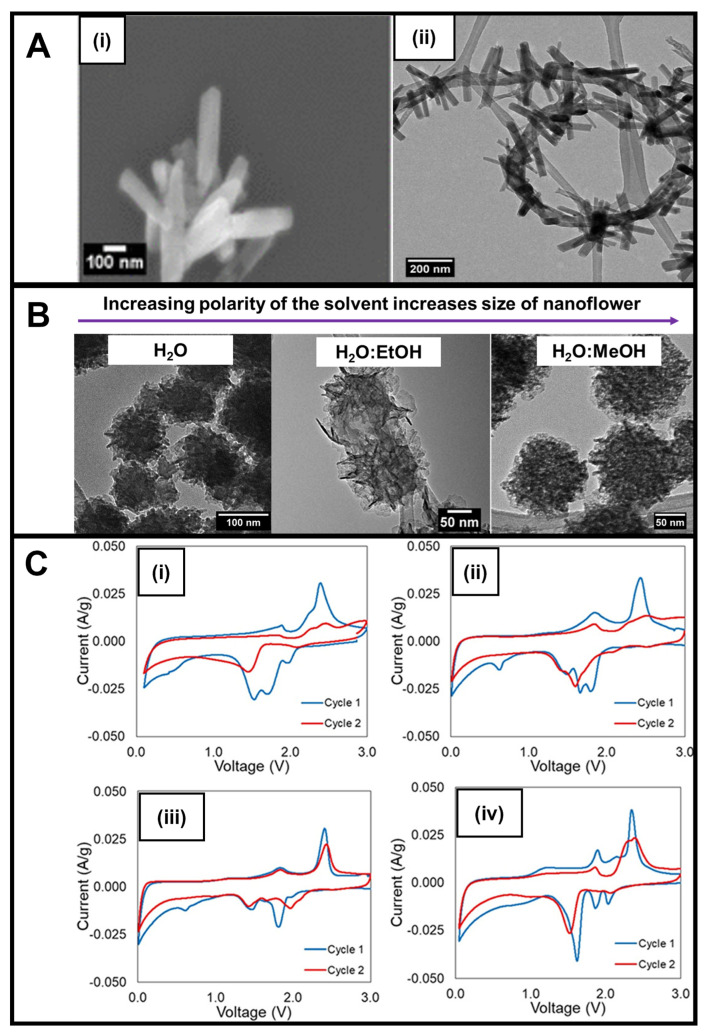
(**A**) (**i**) SEM image of as-prepared VS_4_ nanorods and (**ii**) HRTEM image of as-prepared VS_4_ nanorod/MWNT composites. (**B**) TEM images of VS_4_ as 3D nanoflowers synthesized in the presence of water (NF-W), a water/ethanol mixture, and a water/methanol mixture (NF-M). (**C**) CVs of (**i**) pristine VS_4_ nanorods, (**ii**) annealed VS_4_ nanorods, (**iii**) annealed VS_4_ nanorod/MWNT composites, and (**iv**) VS_4_ nanoflowers [18]. These data sets were obtained at a 0.1 mV/s scan rate between 0.01–0.05 and 3.0 V versus Li/Li^+^. Panels (**A**–**C**) have been adapted with permission from ref. [18]. Copyright 2020 American Chemical Society.

In our lab, whereas 1D nanorods were created using vanadyl acetylacetonate as the vanadium precursor and thioacetamide as the sulfur precursor within a DMF solvent after 10 min of reaction time (Figure 5A(i)), the corresponding 3D nanoflowers could be synthesized by replacing the original vanadium precursor with sodium orthovanadate and substituting DMF with aqueous solvents ranging from a water/ethanol or a pure methanol mixture. In particular, VS_4_ nanoflowers measuring from 100 to 200 nm in diameter were constructed by varying the solvent composition from water onwards to a water/ethanol mixture and ultimately to a water/methanol combination (Figure 5B); increasing solvent polarity led to a corresponding increase in the size of the resulting VS_4_ nanoflowers. Our modification to the microwave synthesis protocol was noteworthy in that the process did not require the use of a conductive carbon substrate and ran at shorter reaction times in a neutral environment while still demonstrating the capability of exerting proper compositional and morphological control [83].

A deliberate approach was used to evaluate the electrochemistry of the samples. Rather than using a planar Cu or Al foil as the current collector, a three-dimensional carbon substrate was utilized [84]. The incorporation of this substrate enabled the evaluation of the as-synthesized material over a wide voltage range with the kinetics of electron and ion transport unimpeded by the electrode design, thereby allowing for evaluation of the core material properties. The experiment was conducted, wherein the lithium electrode served as both the reference and counter electrolyte versus the transition metal sulfide functioning as the working electrode. Regarding the electrochemical testing of these samples, interestingly, unlike most conventional materials, the VS_4_ nanorods did not provide any evidence for the formation of a SEI layer during CV cycling during testing (Figure 5C(i)). Analogous VS_4_ nanorods subjected to thermal annealing even showed a greater cycling stability (Figure 5C(ii)). In the latter case, the annealing process presumably favored crystallite formation, increased the degree of crystallization overall, and diminished the presence of amorphous phases that might otherwise have led to structural disorder and a decrease in Li diffusion.

Upon the addition of MWNTs within the context of an in situ reaction, we were able to form a composite consisting of a distinctive ‘necklace’-like morphology, in which attached, evenly spaced VS_4_ nanorods projected radially outwards from the surface of a circular and winding spatial arrangement of an underlying MWNT backbone (Figure 5A(ii)) [18]. Electrochemical testing of this composite suggested that the addition of the MWNTs via the in situ reaction favorably enhanced both CV cycling stability and conductivity (Figure 5C(iii)). Moreover, the MWNTs themselves also could more readily accommodate for volume changes occurring during the lithiation/delithiation process. Finally, it should be noted that as compared with VS_4_ nanorods, VS_4_ nanoflowers maintained a better enhanced reversibility during CV cycling (Figure 5C(iv)), a finding attributable most likely to an increase in surface area and porosity, which subsequently led to both an improved Li-ion transport and a greater capability to accommodate for volume change [34].

In another study, microwave-synthesized VS_4_ nanosheets were grown as nanospheres, hollow nanospheres, and nanoflowers (with the precise morphology controlled by adjusting the temperature and duration of the microwave heating), and investigated for their performance as anodes in the context of sodium-ion batteries. It was found that the hollow nanospheres yielded the best performance, with a specific capacity of 1226.7 mA h g^−1^ after 100 cycles at 200 mA g^−1^. The long-term cycling capacity was measured to be 1129.6 mA h g^−1^ after 1000 cycles at 2 A g^−1^. This higher performance metric can likely be ascribed to the hollow nanospherical shape, which maximizes surface area-to-volume ratios while offering resistance to volume changes arising from the process of sodium-ion insertion and removal [51].

## 6. Conclusions and Outlook

Metal sulfides are attractive candidates for battery applications due to their favorable electrical conductivity, decent mechanical and thermal stability, relatively low cost, and reasonable electrochemical activity [85,86,87]. As such, the use of microwave-assisted reactions represents a viable, rapid, facile, and reasonably mild methodology with which to produce high-quality, pure metal sulfides in large quantities. These protocols have enabled both chemical and morphological control for the production of metal sulfides, incorporating different sizes and dimensions that are relevant for energy applications. For example, as shown in Table 1, MoS_2_ nanosheets, VS_4_ nanorods, Fe_3_S_4_ nanosheets, and CuS flowers generated through the mediation of microwave-based chemistry have all shown important promise for battery applications.

From the prior literature, it is clear that the effect of varying dimensionality and morphology is significant, although it is difficult to draw any broad, generalizable conclusions. For example, we have found that, with VS_4_, the use of 3D nanoflowers as opposed to 1D nanorods was seen to increase the observed reversibility, presumably due in part to the comparative increase in the number of exposed surface-active sites on the constituent 2D nanosheets of the ‘flower-like’ motifs [18]. By contrast, due to the increased interfacial contact between the metal sulfide and the carbon-based support, In_2_S_3_ particle-graphene composites exhibited an enhanced electrochemical activity as compared with their In_2_S_3_ flower-graphene analogs [44]. Moreover, other unexpected factors, such as the choice of the solution electrolyte, can influence the behavior of metal sulfides incorporated within battery systems; for example, the use of an ether-based electrolyte (as opposed to other electrolyte compositions) can optimize the overall performance and cyclability of certain metal sulfides for battery performance [41,47].

In addition, their associated carbon-based composites, composed of materials in which carbon additives were added to the underlying metal sulfide-based frameworks, such as either In_2_S_3_-nanoparticle-on-sheet or VS_4_ ‘necklace-like’ motifs, exhibited perceptibly enhanced electrochemical performance. Specifically, sulfides combined with MWNTs, graphene, and rGO yielded measurable improvements in both cyclability and capacity, implying the legitimate value of introducing conductive carbon additives as a means of increasing conductivity.

It is worth noting that the intrinsic flexibility of microwave-based techniques suggests the adaptability of this methodology towards fabricating ever more geometrically sophisticated architectures. Regarding the issue of morphological complexity, core@shell motifs are known to increase the stability and capacity of samples for LIB applications, presumably because the core–shell interface creates desirable void spaces that allow for not only the ability to account for volume changes during cycling but also the capability of facilitating electrolyte penetration. As such, Cu@MoS_2_ core–shell nanowires were found to possess a higher reversible capacity of 570.6 mA h g^−1^ after 250 cycles at a current density of 0.5 A g^−1^ as compared with MoS_2_ alone [52].

With respect to the parallel issue of enabling chemical tuning, the example of Mo_1−x_W_x_S_2_ alloy nanoflowers produced from microwave-assisted procedures enabled an improvement in electrochemical performance as compared with either pure WS_2_ or MoS_2_ alone. Specifically, the initial discharge and charge capacities of the Mo_0.5_W_0.5_S_2_ alloy were 774.9 and 635.9 mA h g^−1^, with a high initial Coulombic efficiency (CE) of 82.1%; indeed, the reversible capacity of the alloy was found to increase with measured CE values of 97.0% and 99.4% for the 3rd and 100th cycles, respectively [53]. Furthermore, the Mo_0.5_W_0.5_S_2_ alloy electrode maintained a reversible capacity of 271.9 mA h g^−1^ after 400 cycles. In this case, varying and optimizing the Mo/W ratio led to enhancements in both electronic conductivity and cycling stability, presumably due to the tailorability of the interlayer spacing dimension within these materials, thereby allowing for more effective Li-ion diffusion and volume accommodation.

Moreover, chemical tuning via doping of the sulfur in these materials with other chalcogenide elements using microwave-assisted synthesis procedures is also possible and can boost electrochemical performance as compared with pure sulfides. For example, CuS_0.96_Te_0.04_ nanosheets prepared using microwave-assisted synthesis were found to exhibit excellent performance metrics as a magnesium-ion battery cathode, as these were characterized by a specific capacity of 394.5 mA h g^−1^ at 50 mA g^−1^ current density, as compared with that of 305.4 mA h g^−1^ for pure CuS nanosheets measured under identical conditions. Furthermore, the long-term specific capacity for the CuS_0.96_Te_0.04_ nanosheets yielded a promising 114.8 mA h g^−1^ value after 200 cycles at 500 mA g^−1^. These enhancements in performance are attributed not only to an increase in Mg-ion mobility and diffusion kinetics due to the larger size and polarizability of the Te anions but also to improvements in redox reversibility behavior [54].

While all of this prior work suggests that considerable progress has been made in the investigation of binary metal sulfides for energy storage applications, further research is still necessary for sulfide materials to overcome their current inherent limitations and achieve their full potential. As such, several key issues still need to be tackled and are listed as follows:

(1) In the realm of material science and engineering, crucial chemical strategies associated with addressing the fabrication of tailored heterostructures, doping with heteroatoms, and the directed introduction of defects and vacancies should be more fully explored.
(2) The role of experimental reaction parameters in dictating structure–property-–performance relationships that are relevant to battery performance is a fundamentally difficult challenge in assessing how the desired electrode performance can be informed and ultimately tuned by the rational selection and synthesis of target materials. Probing and optimizing microwave-assisted synthesis methods, with their potential to induce an improved control over nanomaterial composition, nucleation, growth, morphology, and other characteristics, will facilitate all of these important outcomes.
(3) A theoretical understanding of the many factors that contribute to, for instance, electrode performance and stability, coupled with the acquisition of basic mechanistic insights, will be necessary for analyzing and perfecting charge storage behavior. Specifically, computational simulation will enable and strengthen knowledge about relevant reaction mechanisms and kinetics so as to yield greater improvements in battery safety and material optimization.
(4) Finally, although it is beyond the scope of the current review, it is worth pointing out that a number of in situ synchrotron-based characterization techniques, such as operando XRD, ex situ XAS, and XRF, have all been successfully used to assess the simultaneous chemical and physical evolution of sulfide-based systems, including MoS_2_ and CuS within the context of practical, operating batteries, as a means of tracking nuanced changes in their structure, chemistry, and morphology as a function of their electrochemical performance [14,88]. In combination with other structural characterization methods which allow for the monitoring of the evolution of battery components in use, such as in situ TEM [78], these techniques have been employed and will continue to be utilized in terms of elucidating relevant electrochemical mechanisms, analyzing the composition of SEI layers, and providing valuable information about not only possible side reactions but also key electron/ion transport pathways [86].

## Figures and Tables

**Table 1:** Sustained specific capacities measured over multiple cycles of metal sulfide electrode nanomaterials, synthesized through microwave-assisted processes. Unless otherwise noted, capacities were measured in half-cell configurations with the appropriate metal (Li, Na, Mg) as the counter/reference electrode.

Material	Ion Type	Capacity (mA h g^−1^)	Lifespan	Reference
CuS nanosheets	Mg	135	200 cycles @ 200 mA g^–1^	[37]
CuS nanosheet superstructures	Na	347	1000 cycles @ 5 A g^–1^	[38]
CuS nanosphere/CNT composites	Li	437–569	250 cycles @ 400 mA g^–1^	[39]
CuS nanoflower/rGO composites	Li	390	200 cycles @ 500 mA g^–1^	[40]
CuS nanosheet/rGO composites	Na	345	450 cycles @ 1 A g^–1^	[41]
CuS nanoparticles on graphene	Li	348	1000 cycles @ 2 A g^–1^	[26]
ZnS nanoparticle/rGO composites	Na	481	50 cycles @ 100 mA g^–1^	[42]
Co_9_S_8_/rGO composites	Na	346	30 cycles @ 100 mA g^–1^	[43]
In_2_S_3_ nanoflowers on graphene	Li	657	40 cycles @ 70 mA g^–1^	[44]
In_2_S_3_ nanoparticles on graphene	Li	522	100 cycles @ 700 mA g^–1^	[44]
Ni_3_S_2_ and Ni_7_S_6_ nanoparticle/rGO composites	Na	392	50 cycles @ 100 mA g^–1^	[45]
MoS_2_ nanoparticles	Li	544	500 cycles @ 5 A g^–1^	[46]
MoS_2_ nanosheet/CNT-sulfur composites	Li-S	694	200 cycles @ 835 mA g^–1^	[47]
SnS_2_ nanosheet/LEGr composites	Li	664	200 cycles @ 300 mA g^–1^	[48]
WS_2_/graphene composites	Li	714	100 cycles @ 300 mA g^–1^	[49]
WS_2_/graphene composites	Li	534	450 cycles @ 1 A g^–1^	[49]
VS_4_/rGO composites	Li	1144	50 cycles @ 100 mA g^–1^	[50]
VS_4_ hollow nanospheres	Na	1130	1000 cycles @ 2 A g^–1^	[51]
Cu@MoS_2_ core/shell nanowires	Li	571	250 cycles @ 500 mA g^–1^	[52]
Mo_0.5_W_0.5_S_2_ alloy nanoflowers	Li	272	400 cycles @ 1 A g^–1^	[53]
CuS_0.96_Te_0.04_ nanosheets	Mg	115	200 cycles @ 500 mA g^–1^	[54]

## Data Availability

Data is contained within the article itself.
